# Reducing measles mortality in India

**DOI:** 10.7554/eLife.46186

**Published:** 2019-04-09

**Authors:** Anindya Sekhar Bose

**Affiliations:** Immunization Preventable Diseases ProgrammeWorld Health OrganizationKathmanduNepal

**Keywords:** measles, India, mortality, child, immunization, interrupted time series, Human

## Abstract

A large-scale campaign to promote measles vaccinations has substantially reduced the number of children dying from the disease in India.

**Related research article** Wong BKC, Fadel SA, Awasthi S, Khera A, Kumar R, Menon G, Jha P. 2019. The impact of measles immunization campaigns in India using a nationally representative sample of 27,000 child deaths. *eLife* **8**:e43290. doi: 10.7554/eLife.43290

Measles is a highly infectious disease that can lead to serious complications and even death. The measles virus has been around for over 5,000 years and exclusively infects humans (Rota et al., 2016). However, measles can be prevented by a vaccine that is both safe and effective, so it should be possible to eradicate the disease, just as we did with smallpox and are very close to achieving with polio.

The measles vaccine is made of a live but weakened ('live attenuated') form of the measles virus that can induce immunity but does not cause disease, and costs less than a dollar per dose. It was first introduced in the United States in the 1960s, and in September 2016, the World Health Organization (WHO) declared the Americas free of endemic measles. So far, it is the only WHO region to have achieved this status, which, however, was lost in 2018 due to the poor immunization coverage that allowed endemic transmission to be reestablished in Venezuela and to spread to other countries. Vaccine hesitancy behavior, often fueled by misinformation, has also played a part (Dabbagh et al., 2018).

According to WHO estimates, measles vaccinations have prevented around 21 million deaths globally, and nearly 7 million deaths in the WHO region of South East Asia between 2000 and 2017 (Dabbagh et al., 2018). Nevertheless, in 2017, almost one-third (nearly 36,000) of global measles deaths occurred in South East Asia, many of which were in India due to the relativley large number of unvaccinated children.

In 2010, faced with such a high mortality burden from a vaccine-preventable disease, the Indian government introduced a second dose of measles vaccine into its immunization program through a two-pronged approach: routine immunization in 17 states, and mass immunization campaigns targeting children aged between 9 months and 10 years in 14 other states with historically low immunization uptakes. The campaigns were implemented between 2010 and 2013, targeting over 134 million children – a massive undertaking by any standards and one of the largest mass immunization campaigns ever conducted (Centers for Disease Control and Prevention, 2011).

India did not have a sensitive nationwide measles surveillance system that could demonstrate the possible impact of the campaign by providing reliable information about the number of measles cases and deaths before and after the campaign. In some states, such as Gujarat in the west, the number of measles cases went down after the campaign from nearly 1,000 in 2010 to none in 2012. However, the immunization campaign also triggered an interest for a better surveillance system leading to increased case identification, which in turn led to an apparent increase in reported measles cases after the campaign in some states. Therefore, the immunization program was unable to obtain definitive data to measure the impact of the campaign (WHO, 2013; Centers for Disease Control and Prevention, 2011).

Now, in eLife, Prabhat Jha of the University of Toronto and colleagues – including Benjamin Wong as first author – report how they were able to assess the full impact of this enormous immunization campaign (Wong et al., 2019). Wong et al. used data from the Million Death Study, which systematically ascertains the cause of death in a nationally representative sample of deaths in India, including 27,000 child deaths from 1.3 million households between 2005 and 2013 (The Million Death Study Collaborators, 2010; Fadel et al., 2017; Gomes et al., 2017).

The team – which also includes researchers from King George's Medical University, the Ministry of Health and Family Welfare in India, the Post Graduate Institute of Medical Education and Research, and the National Institute of Medical Statistics – used a number of statistical techniques, including an approach called interrupted time series that allowed them to measure the impact of a time-limited intervention (the measles campaign in this case) on events that are continuous (the measles deaths). The basic premise of their study was not to develop a model that could provide estimates of measles mortality: rather, they directly sampled death events before and after the immunization intervention and showed a significant reduction in measles deaths.

In addition, they also compared changes in the occurrence of measles deaths before and after the campaign to changes in child deaths from other causes during the same period. The latter group did not show any reduction, which suggests that the decrease in measles deaths was indeed related to the vaccination campaigns and not to other healthcare improvements.

The analyses revealed that the mass immunization campaigns prevented between 41,000 and 56,000 measles deaths in children (which corresponds to a reduction of 39–57%). In campaign states, mortality rates fell more than in states without a campaign, whereas the number of child deaths from other causes did not fall. The results reported by Wong et al. should help public health officials in India and elsewhere to take informed decisions in their efforts to control or eliminate measles cases and deaths using mass immunization campaigns.
